# The Basis Pathology and Specific Treatment Diphtheria

**Published:** 1885-05

**Authors:** 


**The Basic Pathology and Specific Treatment of Diphtheria, Typhoid, Zymotic, Septic Scorbutic and Putrescent Diseases Generally.** By GEO. J. ZIEGLES, M. D. Philadelphia: Geo. J. Ziegles, M. D. 1884. Price, $2.00.

What the author aims to present in this work, under the term "Basic Pathology and Specific Treatment," we give in his words, taken from the preface: "I have herein tried to show that all the varied and complex diseases are dependent upon or complicated with one common basic alkaline pathogenic factor, mostly the volatile organic alkali, ammonia, incidental to all forms of life, etc., for the successful treatment of which this primal morbific factor must be decomposed, neutralized or removed by acidulous, autalkaline, resolving and counteracting agents, etc." We have little to say in regard to this theory, for such it is. The author presents it in a very plausible form, and readers can only judge of its merits by perusing the book.

